# Agents for fluorescence-guided glioma surgery: a systematic review of preclinical and clinical results

**DOI:** 10.1007/s00701-016-3028-5

**Published:** 2016-11-22

**Authors:** Joeky T. Senders, Ivo S. Muskens, Rosalie Schnoor, Aditya V. Karhade, David J. Cote, Timothy R. Smith, Marike L. D. Broekman

**Affiliations:** 1 Department of Neurosurgery, Brain Center Rudolf Magnus, University Medical Center Utrecht, Heidelberglaan 100, 3584 CX Utrecht, The Netherlands; 2 Department of Neurosurgery, Cushing Neurosurgery Outcomes Center, Brigham and Women’s Hospital, Harvard Medical School, 15 Francis Street, Boston, MA 02115 USA

**Keywords:** Fluorescence-guided surgery, Glioma, Neurosurgery, 5-ALA, Fluorescein

## Abstract

**Background:**

Fluorescence-guided surgery (FGS) is a technique used to enhance visualization of tumor margins in order to increase the extent of tumor resection in glioma surgery. In this paper, we systematically review all clinically tested fluorescent agents for application in FGS for glioma and all preclinically tested agents with the potential for FGS for glioma.

**Methods:**

We searched the PubMed and Embase databases for all potentially relevant studies through March 2016. We assessed fluorescent agents by the following outcomes: rate of gross total resection (GTR), overall and progression-free survival, sensitivity and specificity in discriminating tumor and healthy brain tissue, tumor-to-normal ratio of fluorescent signal, and incidence of adverse events.

**Results:**

The search strategy resulted in 2155 articles that were screened by titles and abstracts. After full-text screening, 105 articles fulfilled the inclusion criteria evaluating the following fluorescent agents: 5-aminolevulinic acid (5-ALA) (44 studies, including three randomized control trials), fluorescein (11), indocyanine green (five), hypericin (two), 5-aminofluorescein-human serum albumin (one), endogenous fluorophores (nine) and fluorescent agents in a pre-clinical testing phase (30). Three meta-analyses were also identified.

**Conclusions:**

5-ALA is the only fluorescent agent that has been tested in a randomized controlled trial and results in an improvement of GTR and progression-free survival in high-grade gliomas. Observational cohort studies and case series suggest similar outcomes for FGS using fluorescein. Molecular targeting agents (e.g., fluorophore/nanoparticle labeled with anti-EGFR antibodies) are still in the pre-clinical phase, but offer promising results and may be valuable future alternatives.

## Introduction

Radical surgical resection is the surgical treatment of choice for gliomas [95, 102]. Balancing maximum cytoreduction with preservation of healthy brain tissue is complicated by the infiltrative nature of these tumors [88, 96]. Fluorescent agents are increasingly being tested and used to distinguish tumor from normal parenchyma thus improving surgical resection while sparing healthy brain tissue [17, 57, 59, 76, 119]. The only fluorescent agent that has been tested in a multi-center randomized controlled trial (RCT) and the only agent currently approved for resection of high-grade gliomas (HGGs) in Europe, Canada, and Japan is 5-aminolevulinic acid (5-ALA) [67]. In clinical studies, the use of 5-ALA for fluorescence-guided surgery (FGS) has been shown to increase the rate of gross-total resection (GTR) and the length of progression-free survival (PFS) [99]. As a relatively nascent innovation, FGS for glioma is still limited by many factors, which depend on the fluorescent agent used. In this systematic review, we assess the use of all clinically tested fluorescent agents in FGS for glioma. Furthermore, we evaluate all pre-clinically tested fluorescent agents with the potential for FGS for glioma.

## Methods

### Search strategy

We performed an extended search in PubMed and Embase databases according to the Preferred Reporting Items for Systematic Reviews and Meta-Analyses (PRISMA) guidelines on March 21, 2016. We included all articles investigating the use of fluorescent agents for identification or resection of glioma tumor cells in both the clinical and pre-clinical settings. This review is restricted to published literature. Only papers written in English and Dutch were included. The search was not limited by date of publication. We did not include pre-clinical studies on 5-ALA and fluorescein, as these agents have been used extensively in the clinical setting. The search syntax is available in Table 1. The systematic search was complemented by additional citations identified by hand searching the bibliographies of the papers retrieved by the electronic search. The title and abstracts of retrieved studies were screened, and full texts of potentially suitable articles were read by three authors (JS, RS, IM). Disagreements were resolved by discussion.

**Table 1:** Search syntax

PubMed search accessed on 03–21–2016	Embase search accessed on 03–21–2016
((“Fluorescent Dyes”[Mesh] OR pigments [Title/Abstract] OR pigment [Title/Abstract] OR stains [Title/Abstract] OR stain [Title/Abstract] OR fluorophores [Title/Abstract] OR fluorophore [Title/Abstract] OR contrast agents [Title/Abstract] OR contrast agent [Title/Abstract] OR dye [Title/Abstract] OR fluorescent [Title/Abstract] OR fluorescence [Title/Abstract] OR fluorochromes [Title/Abstract] OR fluorogenic substrate [Title/Abstract] OR coloring agents [Title/Abstract] OR coloring agent [Title/Abstract] OR luminescent agents [Title/Abstract] OR luminescent agent [Title/Abstract] OR 5-ALA [Title/Abstract] OR 5-aminolevulinic acid [Title/Abstract]) AND (“Glioma”[Mesh] OR glioma [Title/Abstract] OR gliomas [Title/Abstract] OR GBM [Title/Abstract] OR glioblastoma [Title/Abstract] OR brain tumor [Title/Abstract] OR brain tumors [Title/Abstract] OR brain tumour [Title/Abstract] OR brain tumours [Title/Abstract] OR brain cancer [Title/Abstract]) AND (“Neurosurgical Procedures”[Mesh] OR operation [Title/Abstract] OR surgery [Title/Abstract] OR surgical [Title/Abstract] OR neurosurgery [Title/Abstract] OR resection [Title/Abstract]))	(‘fluorescent dye’/exp OR pigments:ti:ab OR pigment:ti:ab OR stains:ti:ab OR stain:ti:ab OR fluorophores:ti:ab OR fluorophore:ti:ab OR (contrast AND agents):ti:ab OR (contrast AND agent):ti:ab OR dye:ti:ab OR fluorescent:ti:ab OR fluorescence:ti:ab OR fluorochromes:ti:ab OR (fluorogenic AND substrate):ti:ab OR (coloring AND agents):ti:ab OR (coloring AND agent):ti:ab OR (luminescent AND agents):ti:ab OR (luminescent AND agent):ti:ab OR 5-ALA:ti:ab OR (5-aminolevulinic AND acid):ti:ab) AND (‘glioma’/exp OR glioma:ti:ab OR gliomas:ti:ab OR GBM:ti:ab OR glioblastoma:ti:ab OR (brain AND tumor):ti:ab OR (brain AND tumors):ti:ab OR (brain AND tumour):ti:ab OR (brain AND tumours):ti:ab OR (brain AND cancer):ti:ab) AND (‘neurosurgery’/exp OR operation:ti:ab OR surgery:ti:ab OR surgical:ti:ab OR neurosurgery:ti:ab OR resection:ti:ab)

### Data extraction

The following data were extracted from selected papers: year of publication, name of first author, fluorescent agent tested, study design, number of patients, tumor grade, GTR rate, sensitivity and specificity of the fluorescent agent for tumor tissue, tumor-to-normal ratio (TNR) of the fluorescent signal, median survival, progression-free survival (PFS), and incidence of adverse events. GTR was defined as no residual enhancement on post-operative magnetic resonance imaging (MRI). Overall survival and PFS was quantified in months. Among the included studies, histological accuracy was quantified in two ways. Some studies collected tissue samples near the tumor margin from fluorescent and non-fluorescent areas for histopathological examination and calculated the sensitivity and specificity of distinguishing tumor from healthy brain tissue. Others measured the fluorescent signal intensity from tumor and brain tissue and calculated a TNR. We considered grade I and II tumors as low-grade gliomas (LGGs) and grade III and IV gliomas as high-grade gliomas (HGGs) according to the 2016 World Health Organization (WHO) classification of tumors of the central nervous system [60].

## Results

**Fig. 1:**
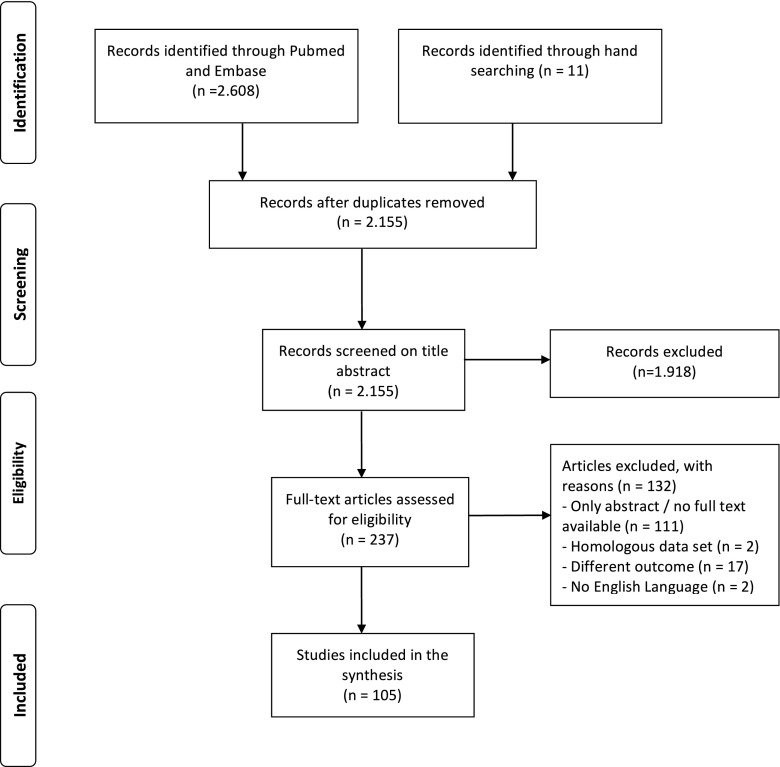
Flowchart depicting study selection

We identified 2155 studies in PubMed and Embase after duplicates were removed. After screening by title and abstract, 237 studies remained for full-text review. Of these, we included 105 studies describing the use of clinically or pre-clinically tested fluorescent agents for application in FGS for glioma (Fig. 1). Detailed characteristics of all 105 studies included in this review are available in Table 2. Three studies were randomized clinical trials, of which two had partially the same data set. Three studies were meta-analyses. The other clinical studies were retrospective or prospective cohort studies, or case series. Preclinical studies included human or animal ex vivo studies, animal in vivo studies, or in vitro studies.

**Table 2:** Overview of all studies

Year	Fluorescent agent	Study design	No. of cases	Tumor grade of patients	Control group	GTR rate (%)	TNR	Median survival (mo)	PFS (mo)	6-PFS (%)
Stummer et al. 2000 [98]	5-ALA	Case series	52	GBM	No	63	–	3	–	–
Stummer er al. 2006 [99]	5-ALA	RCT	322	HGG	Yes	65	–	15	5	41
Eljamel et al. 2008 [26]	5-ALA	RCT	27	GBM	No	–	–	12	9	–
Hefti et al. 2008 [41]	5-ALA	Case series	74	HGG	No	–	–	–	–	–
Nabavi et al. 2009 [66]	5-ALA	Case series	36	HGG	No	–	–	–	–	–
Feigl et al. 2010 [31]	5-ALA	Case series	18	HGG	No	64	–	–	–	83
Ewelt et al. 2011 [28]	5-ALA	Case series	17	HGG	No	–	–	–	–	–
Ewelt et al. 2011 [28]	5-ALA	Case series	13	LGG	No	–	–	–	–	–
Floeth et al. 2011 [33]	5-ALA	Case series	21	HGG	No	–	–	–	–	–
Floeth et al. 2011 [33]	5-ALA	Case series	17	LGG	No	–	–	–	–	–
Diez Valle et al. 2011 [25]	5-ALA	Case series	28	GBM	No	83	–	16	–	68
Roberts et al. 2011 [81]	5-ALA	Case series	11	GBM	No	–	–	–	–	–
Stummer et al. 2011b [97]	5-ALA	Case series	243	HGG	No	–	–	16	–	–
Stummer et al. 2011a [101]	5-ALA	RCT	349	HGG	Yes	–	–	14	–	46
Idoate et al. 2011 [44]	5-ALA	Case series	30	GBM	No	83	–	–	–	–
Sanai et al. 2011 [84]	5-ALA	Case series	10	LGG	No	–	–	–	–	–
Valdes et al. 2011 [110]	5-ALA	Cohort	14	LGG&HGG	Yes	–	–	–	–	–
Panciani et al. 2012 [74]	5-ALA	Case series	23	GBM	No	–	–	–	–	–
Cortnum et al. 2012 [16]	5-ALA	Case series	13	HGG	No	70	–	–	–	–
Eyopuglu et al. 2012 [30]	5-ALA	Case series	37	HGG	No	71–100	–	–	–	–
Schucht et al. 2013 [87]	5-ALA	Case series	56	GBM	No	89^b^	–	–	–	–
Widhalm et al. 2013 [113]	5-ALA	Case series	59	LGG&HGG	No	–	–	–	–	–
Della puppa et al. 2013 [22]	5-ALA	Case series	31	HGG	No	74	–	–	–	–
Slotty et al. 2013 [94]	5-ALA	Cohort	253	GBM	Yes	49	–	20	–	–
Aldave et al. 2013 [3]	5-ALA	Case series	118	HGG	No	62	–	21	–	–
Diez Valle et al. 2014 [24]	5-ALA	Cohort	251	HGG	Yes	67	–	–	–	69
Roder et al. 2014 [82]	5-ALA	Cohort	66	GBM	Yes	46	–	–	–	–
Belloch et al. 2014 [6]	5-ALA	Case series	21	HGG	No	71^b^	–	–	–	–
Schucht et al. 2014 [89]	5-ALA	Case series	72	GBM	No	73	–	–	–	–
Coburger et al. 2014 [13]	5-ALA	Case series	34	HGG	No	–	–	–	–	–
Piquer et al. 2014 [75]	5-ALA	Case series	38	HGG	No	61	–	–	–	–
Stummer et al. 2014 [100]	5-ALA	Case series	33	HGG	No	–	–	–	–	–
Barbagallo et al. 2015 [5]	5-ALA	Cohort	50	HGG	Yes	97	–	–	–	–
Coburger et al. 2015 [14]	5-ALA	Cohort	33	GBM	Yes	100	–	18	6	–
Cordova et al. 2015 [15]	5-ALA	Case series	30	GBM	No	–	–	–	–	29
Gessler et al. 2015 [34]	5-ALA	Case series	32	GBM	No	97	–	19	14	–
Haj-Josseini et al. 2015 [37]	5-ALA	Case series	30	HGG	No	–	–	–	–	–
Hickmann et al. 2015 [42]	5-ALA	Cohort	58	HGG	Yes	57^c^	–	20	12	–
Noell et al. 2015 [70]	5-ALA	Case series	29	HGG	No	25	–	19	–	47
Schatlo et al. 2015 [85]	5-ALA	Case series	200	HGG	No	–	–	–	–	–
Szmuda et al. 2015 [105]	5-ALA	Case series	21	HGG	No	57^c^	–	–	–	–
Valdes et al. 2015 [109]	5-ALA	Case series	12	LGG	No	–	–	–	–	–
Yamada et al. 2015 [115]	5-ALA	Case series	99	HGG	No	–	–	–	–	–
Hauser et al. 2016 [40]	5-ALA	Case series	13	GBM	No	77	–	14	–	31
Quick-Weller et al. 2016 [78]	5-ALA	Case series	7	GBM	No	–	–	–	–	–
Teixidor et al. 2016 [106]	5-ALA	Case series	85	HGG	No	54^b^	–	14	7	58
Moore et al. 1948 [65]	Fluorescein	Case series	12	LGG&HGG	No	–	–	–	–	–
Shinoda et al. 2003 [92]	Fluorescein	Cohort	32	GBM	Yes	84^b^	–	15	–	–
Koc et al. 2008 [52]	Fluorescein	Cohort	37	GBM	Yes	83	–	11	–	–
Chen et al. 2012 [11]	Fluorescein	Cohort	22	LGG&HGG	Yes	80^b^	–	–	7	–
Kuroiwa et al. 1998 [54]	Fluorescein	Case series	10	HGG	No	100	–	–	–	–
Okuda et al. 2012 [71]	Fluorescein	Case series	10	GBM	No	100	–	–	–	–
Schebesch et al. 2013 [86]	Fluorescein	Case series	35	LGG&HGG	No	80	–	–	–	–
Acerbi et al. 2014 [1]	Fluorescein	Case series	20	HGG	No	80	–	–	–	72
Diaz et al. 2015 [23]	Fluorescein	Case series	12	HGG	No	100	–	–	–	–
Hamancioglu et al. 2016 [38]	Fluorescein	Case series	28	HGG	No	79^b^	–	–	–	–
Martirosyan et al. 2016 [64]	Fluorescein	Case series	74	LGG&HGG	No	–	–	–	–	–
Hansen et al. 1993 [39]	ICG	Preclinical	–	–	No	–	–	–	–	–
Haglund et al. 1994 [36]	ICG	Preclinical	22	–	No	–	–	–	–	–
Haglund et al. 1996 [35]	ICG	Case series	9	LGG&HGG	No	–	–	–	–	–
Martirosyan et al. 2011 [63]	ICG	Preclinical	30	–	No	–	–	–	–	–
Eyupoglu et al. 2015 [29]	ICG	Case series	3	HGG	No	–	–	–	–	–
Kremer et al. 2009 [53]	AFL-HSA	Case series	13	HGG	No	69	–	–	–	–
Noell et al. 2011 [70]	Hypericin	Preclinical	16	–	No	–	19.8	–	–	–
Ritz et al. 2012 [80]	Hypericin	Case series	5	HGG	No	–	–	–	–	–
Lin et al. 2001 [58]	Endogenous	Case series	26	LGG&HGG	No	–	–	–	–	–
Toms et al. 2005 [107]	Endogenous	Case series	24	LGG&HGG	No	–	–	–	–	–
Marcu et al. 2004 [62]	Endogenous	Preclinical	6	–	No	–	–	–	–	–
Yong et al. 2006 [117]	Endogenous	Case series	31	LGG&HGG	No	–	–	–	–	–
Butte et al. 2011 [9]	Endogenous	Case series	24	LGG&HGG	No	–	–	–	–	–
Leppert et al. 2006 [56]	Endogenous	Preclinical	–	–	No	–	–	–	–	–
Kantelhardt et al. 2009 [50]	Endogenous	Preclinical	–	–	No	–	–	–	–	–
Riemann et al. 2012 [79]	Endogenous	Preclinical	–	–	No	–	–	–	–	–
Kantelhardt et al. 2016 [49]	Endogenous	Case series	8	–	No	–	–	–	–	–
Veiseh et al. 2007 [111]	Fluorophore	Preclinical	22	–	No	–	–	–	–	–
Lanzardo et al. 2011 [55]	Fluorophore	Preclinical	4	–	No	–	–	–	–	–
Yan et al. 2011 [116]	Fluorophore	Preclinical	–	–	No	–	1.6	–	–	–
Agnes et al. 2012 [2]	Fluorophore	Preclinical	–	–	No	–	–	–	–	–
Cutter et al. 2012 [20]	Fluorophore	Preclinical	3	–	No	–	–	–	–	–
Huang et al. 2012 [43]	Fluorophore	Preclinical	–	–	No	–	16.3–79.7	–	–	–
Burden-Gulley et al. 2013 [7]	Fluorophore	Preclinical	–	–	No	–	11.7–19.8	–	–	–
Ma et al. 2014 [61]	Fluorophore	Preclinical	–	–	No	–	–	–	–	–
Crisp et al. 2014 [18]	Fluorophore	Preclinical	14	–	No	–	7.8	–	–	–
Fenton et al. 2014 [32]	Fluorophore	Preclinical	20	–	No	–	–	–	–	–
Butte et al. 2014 [8]	Fluorophore	Preclinical	–	–	No	–	–	–	–	–
Qiu et al. 2015 [77]	Fluorophore	Preclinical	36	–	No	–	–	–	–	–
Swanson et al. 2015 [104]	Fluorophore	Preclinical	35	–	Yes	–	9.28	–	–	–
Warram et al. 2015 [112]	Fluorophore	Preclinical	5	–	No	–	–	–	–	–
Antaris et al. 2016 [4]	Fluorophore	Preclinical	5	–	No	–	5.50	–	–	–
Davis et al. 2010 [21]	Fluorophore	Preclinical	15	–	No	–	–	–	–	–
Sexton et al. 2013 [91]	Fluorophore	Preclinical	4	–	No	–	–	–	–	–
Irwin et al. 2014 [45]	Fluorophore	Preclinical	8	–	No	–	–	–	–	–
Kantelhardt et al. 2010 [48]	Nanoparticle	Preclinical	2	–	No	–	200–1000	–	–	–
Seekell et al. 2013 [90]	Nanoparticle	Preclinical	6	–	No	–	–	–	–	–
Kircher et al. 2003 [51]	Nanoparticle	Preclinical	5	–	No	–	–	–	–	–
Trehin et al. 2006 [108]	Nanoparticle	Preclinical	14	–	No	–	–	–	–	–
Cai et al. 2006 [10]	Nanoparticle	Preclinical	–	–	Yes	–	–	–	–	–
Jackson et al. 2007 [46]	Nanoparticle	Preclinical	–	–	No	–	–	–	–	–
Orringer et al. 2009 [73]	Nanoparticle	Preclinical	–	–	No	–	–	–	–	–
Jiang et al. 2013 [47]	Nanoparticle	Preclinical	18	–	Yes	–	–	–	–	–
Ni et al. 2014 [68]	Nanoparticle	Preclinical	–	–	No	–	–	–	–	–
Zhou et al. 2015 [120]	Nanoparticle	Preclinical	6	–	Yes	–	–	–	–	–
Cui et al. 2015 [19]	Nanoparticle	Preclinical	344	–	No	–	–	–	–	–
Roller et al. 2015 [83]	Nanoparticle	Preclinical	10	–	No	–	–	–	–	–
Zhao et al. 2013 [119]	5-ALA	Meta-analysis	10^a^	–	–	–	–	–	–	–
Su et al.2014 [103]	Multiple	Meta-analysis	12^a^	–	–	72	–	–	5	–
Eljamel et al. 2015 [27]	5-ALA	Meta-analysis	20^a^	–	–	75	–	–	8	–

*5-ALA* δ-Aminolevulinic acid, *6-PFS* 6-month progression-free survival, *AFL-HSA* 5-aminofluorescein labeled to human serum albumin, *GTR* gross-total resection, *ICG* indocyanine green, *mo* months, *no*. number, *PFS* progression-free survival, *TNR* tumor-to-normal ratio, − not specified

^a^Number of studies included in the meta-analysis

^b^Assessment of postoperative MRI up to > 72 h after surgery

^c^Timing of assessment of postoperative MRI not reported

### Clinically tested fluorescent agents

Sixty-four studies describe the clinical use of fluorescent agents [1, 3, 5, 6, 9, 11, 13–16, 22–26, 28–31, 33–35, 37, 38, 40–42, 44, 49, 52–54, 58, 64–66, 69, 71, 74, 75, 78, 80–82, 84–87, 89, 92, 94, 97–101, 105–107, 109, 110, 113, 115, 118]. Three ways of labeling tumor cells were identified in the literature: (1) passive, (2) metabolic, and (3) molecular labeling. Passive labeling occurs when enhanced permeability and retention allow exogenous agents to accumulate at the tumor site. The damaged blood–brain barrier (BBB) allows exogenous agents (e.g., fluorescein or ICG) to concentrate in glioma tissue [67]. Metabolic fluorescent agents (e.g., 5-ALA) are internalized and metabolized intracellularly [99]. Molecular targeting refers to the binding of agents to specific molecules on the cell surface of the tumor cell. A popular target is the epidermal growth factor receptor (EGFR) [67].

### 5-aminolevulinic acid (5-ALA)

5-ALA is a metabolic targeting agent and the natural precursor of the fluorescent protoporphyrin (PpIX) in the heme synthesis pathway. Ferrochelatase converts PpIX into heme intracellularly by adding a Fe2 + −ion. In glioma cells, ferrochelatase is downregulated. Therefore, these cells accumulate PpIX to a fluorescently detectable level when this pathway is overloaded with exogenous 5-ALA. PpIX absorbs light between 375 and 440 nm and emits light between 640 and 710 nm [119].

Forty-four clinical studies described the use of 5-ALA for glioma surgery [3, 5, 6, 13–16, 22, 24–26, 28, 30, 31, 33, 34, 37, 40–42, 44, 66, 69, 74, 75, 78, 81, 82, 84, 85, 87, 89, 94, 97–101, 105, 106, 109, 110, 113, 115], of which only six studies included LGGs [28, 33, 84, 109, 110, 113]. 5-ALA is the only fluorescent agent that has been tested in an RCT. Three RCTs compared FGS with 5-ALA to surgery without fluorescent guidance, with two studies including partially the same patients [26, 99, 101]. The study by Stummer et al. showed a higher GTR rate for the 5-ALA group compared to the group that was operated without 5-ALA (65 vs. 35%) [99]. In observational studies, the rate of GTR after 5-ALA administration ranges from 25 to 94.3% for HGGs [3, 5, 6, 14, 16, 22, 24, 25, 30, 31, 34, 40, 42, 44, 69, 75, 82, 87, 89, 94, 98, 99, 105, 106, 113]. However, studies that included a control group all confirm a significantly higher GTR rate in the FGS group [14, 24, 82, 94, 99]. Two RCTs reported an increased PFS (8.6 vs. 4.8 months) [26] or increased rate of 6-month PFS (46 vs. 28%) [101]. Regarding extending overall survival by using 5-ALA during neurosurgical resection, results vary between non-significant (14–15 vs. 13–14 months) [99, 101] and significant (12 vs. 6 months) [26] survival benefits.

Observational studies show a broad range regarding sensitivity and specificity in discriminating HGG tissue from healthy brain tissue [13, 25, 28, 33, 34, 37, 40, 41, 74, 81, 98, 105, 110, 113, 115]. For discriminating glioblastoma multiforme (GBM) tissue from healthy brain tissue, the sensitivity and specificity ranged from 70 to 95% and 43 to 100%, respectively [25, 34, 74, 98]. All four studies that included both LGG and HGG patients reported a lower sensitivity and specificity in LGGs [28, 33, 110, 113]. To increase the accuracy of LGGs, FGS was combined with intra-operative confocal microscopy [84] or an intraoperative probe for quantitative fluorescence measurement [109]. Other intraoperative techniques used to increase the extent of glioma resection are photodynamic therapy (PDT) [26], iMRI [14, 30, 34, 40, 78, 85], intra-operative CT [5], exoscope imaging [6, 75], fluorescence spectrometry [37, 100], confocal microscopy [84], and intraoperative mapping [22, 89].

Three meta-analyses have been performed to evaluate the literature on 5-ALA [27, 103, 120]. GTR and PFS were improved in all meta-analyses that compared 5-ALA with conventional white-light surgery. A significant difference in overall survival was reported in two meta-analyses [27, 120]. One meta-analysis reported no significant difference in overall survival [103], however, this meta-analysis also included studies on fluorescein for overall survival. The mean sensitivity and specificity in distinguishing tumor from healthy brain tissue ranged between 83 and 87% and 89 and 91% in all three meta-analyses, respectively.

Only the RCT by Stummer et al. 2011 found a significant difference in the incidence of adverse effects. The 5-ALA group had more frequent deterioration at the National Institute of Health Stroke Scale (NIH-SS) at 48 h after surgery [101]. Other reported adverse effects of 5-ALA include nausea, mild hypotension, elevated liver enzymes, and photosensitivity up to 48 h post administration [12, 119].

### Fluorescein

Eleven papers described the use of fluorescein as a fluorescent agent in glioma surgery [1, 11, 23, 38, 52, 54, 64, 65, 71, 86, 92]. All were observational studies including patients with HGG. Only three studies included patients with LGG [11, 64, 86]. Fluorescein is a passive targeting agent commonly used for ophthalmic examinations of the retina [67]. Interestingly, as early as in 1948, a study demonstrated a positive predictive value of 96% in locating brain tumors [65]. Fluorescein is administered intravenously at induction of anesthesia or at time of opening the dura. It is excited at a wavelength of 460–500 nm and has an emission spectral range of 540–690 nm. As this is within the spectrum of visible light, fluorescein is used with [1, 23, 27, 38, 54, 71, 86, 120] or without a filter on the surgical microscope [11, 52, 64, 65, 92].

Nine studies showed that upon administration of fluorescein, GTR can be achieved in 79–84% of patients [1, 11, 23, 38, 52, 54, 71, 86, 92]. Studies comparing the use of fluorescein to conventional white light surgery showed a GTR-rate of 30–55% in the latter group [11, 52, 92]. The use of a special filter integrated into the microscope resulted in an even higher GTR rate of 80–100%; this integrated filter allowed for more accurate delineation at the tumor border and required less fluorescein for visualization (3–8 mg/kg with filter instead of 20 mg/kg without filter in the microscope) [1, 23, 27, 38, 54, 71, 86, 120].

The effect of fluorescein on survival has been evaluated by four groups. Chen et al. found an increase in PFS (7.4 vs. 5.4 months) [11]. Others did not find an increase in overall survival [52, 92] or did not compare with a control group [1].

Three papers reported on the presence of tumor cells in fluorescein negative areas [11, 54, 92]. Others reported that fluorescein identifies tumor tissue with a sensitivity and specificity of 82–94% and 90–91%, respectively [1, 23, 64]. To enhance histological accuracy, Martirosyan et al. explored the use of confocal microscopy in combination with fluorescein [64]. This technique makes use of a handheld probe containing a miniature scanner. The scanner can be placed in direct contact with the tissue of interest and can be visualized on a connected external monitor. The imaging field has a diameter of 0.5 mm. With the integrated depth actuator in the probe, the surgeon can focus on a specific depth beneath the contact plane ranging from 0 to 500 μm. Confocal microscopy with fluorescein is able to visualize individual invading cells at the tumor margin and even subcellular histological features. A sensitivity and specificity of 91 and 94%, respectively, was reported in distinguishing tumor from healthy brain tissue [64].

Studies that included patients with LGG did not stratify for tumor grade. One study reported that visualization was less obvious in LGGs or in recurrent tumors (that had previously been resected or irradiated), due to accumulation of scar tissue. In a survey of five neurosurgeons, fluorescein was rated as ‘helpful’ in visualizing gliomas in 80% of the cases [86].

Side effects of fluorescein include yellow coloration of skin, mucosa, and urine up to 24 h after surgery, generally seen only after high-dose (20 mg/kg) fluorescein [65, 71, 92]. No side effects were detected with low-dose (2–8 mg/kg) fluorescein [1, 23, 38, 54, 86]. Anaphylactic reactions to fluorescein have been reported [117].

### Indocyanine green (ICG)

Two clinical and three pre-clinical studies reported on the use of ICG for glioma surgery [29, 35, 36, 39, 63, 93]. ICG has a peak emission at 820 nm. This near-infrared (NIR) spectrum allows visualization of deeper structures than does visible wavelength. ICG works as a passive targeting agent and depends on the breakdown of the BBB to concentrate at the tumor site. It is already used for several clinical applications, including determining cardiac output, ascertaining hepatic function and liver blood flow, and implementing ophthalmic angiography. ICG is administered intravenously before resection or afterwards to visualize remaining tumor tissue [67].

No articles evaluated the rate of GTR or survival in patients treated with ICG. In rat glioma models, ICG shows an underestimation of 1 mm of the histological tumor border [39] and a sensitivity and specificity of 90 and 93%, respectively [36]. In humans, low-dose ICG (1–2 mg/kg) combined with a filter microscope revealed remaining tumor tissue after resection. Detection was superior in high-grade compared to low-grade gliomas [35]. In a recent case series that combined both fluorescent agents for GBM resection, three tumor zones could be distinguished from the center to the margin of the tumor: a central zone that was stained by both compounds, a zone that was stained by only ICG and not 5-ALA, and the most peripheral zone that contained tumor cells but was not stained by any of the compounds. This suggests that ICG is superior to 5-ALA in staining tumor tissue with a low cell density [29]. Confocal microscopy visualized individual invading tumor cells in peritumoral tissue in a GBM mouse model, and subcellular structures correlated with histological features. The NIR wavelength allowed an imaging plan depth of >350 μm [63].

No complications or adverse effects of ICG were mentioned in these studies. Anaphylactic reactions to ICG have been reported [72].

### 5-aminoflurescein human serum albumin

One case series assessed the passive tumor-targeting agent 5-aminofluorescein (AFL) labeled to human serum albumin (HSA) (excitation 495 nm, emission 535 nm). FGS with AFL-HSA in 13 patients with HGG resulted in a GTR rate of 69%. No phototoxic, allergic, or other side effects related to AFL-HSA were observed [53].

### Hypericin

One case series and one pre-clinical study assessed hypericin, a passive tumor-targeting agent. Hypericin (excitation 415–495 nm; emission 590–650 nm) is intravenously administered in patients undergoing surgery for HGG. Tissue samples from fluorescent and non-fluorescent areas showed a sensitivity and specificity in distinguishing human brain and tumor tissue of 91–94% and 90–100%, respectively. No side effects were observed [80]. In an animal study, rats were implanted with GBM cells and intravenously injected with hypericin. The accumulation of hypericin in the brain was studied ex vivo under a fluorescence microscope. The tumor-to-normal ratio (TNR) was 19.8, after correction for auto-fluorescence [70]. No adverse effects were observed.

### Endogenous fluorophores

Endogenous fluorophores (e.g., NAD(P) H, FAD, and collagen) in brain and tumor tissue can emit fluorescent signals after excitation. Nine studies, five of which were clinical, assessed the use of endogenous fluorophores [9, 49, 50, 56, 58, 62, 79, 107, 118]. Four case series evaluated endogenous fluorophores by using optical spectroscopy [9, 58, 107, 118] and one case series used multiphoton excitation tomography [49]. With optical spectroscopy, a fiber optic probe is placed against the tissue of interest to detect the fluorescent signal. An algorithm then distinguishes brain and tumor tissue [9]. Two studies including both patients with HGG and with LGG achieved a sensitivity and specificity in discriminating infiltrative tumor margin and healthy tissue of 94–100% and 76–93%, respectively [58, 107]. The decrease of fluorescent signal in time provides additional information. Adding this extra dimension to the algorithm, sensitivity and specificity in discriminating LGG from normal brain tissue were 90–100% and 98–100%, respectively. Due to necrosis and a high degree of heterogeneity, however, the sensitivity and specificity for HGG were 47–95% and 94–96%, respectively [9, 118].

Multiple excitation beams from different angles allow excitation wavelengths to be in the infrared spectrum. This reduces phototoxicity, light scattering, and artifacts from blood, and increases the penetration depth. Excitation only occurs when two low-energy photons are simultaneously absorbed by the fluorophore where the laser beams coincide, reducing the amount of background signal. Kantelhardt et al. were the first to use multiphoton excitation tomography intra-operatively in humans, and reported the ability to differentiate between tumor and brain tissue on cellular and subcellular levels [49]. No adverse effects were observed.

#### Pre-clinically tested fluorescent agents

Thirty studies described the results of fluorescent agents in a pre-clinical phase (Table 2) [2, 4, 7, 8, 10, 18–21, 32, 43, 45–48, 51, 55, 61, 68, 73, 77, 83, 90, 91, 104, 108, 111, 112, 116, 121]. Within this group of fluorescent agents, a broad distinction could be made between molecular fluorophores and nanoparticles. Molecular fluorophores are small-sized molecules with fluorescent properties. ICG and fluorescein are examples of clinically tested organic molecular fluorophores [76]. Nanoparticles are structures of nanometer size (1–100 nm). Depending on their structure, nanoparticles can contain optical properties or obtain optical properties by labeling with fluorophores. Targeting properties of both fluorophores and nanoparticles are tunable by adding targeting peptides [76]. Due to their larger size, nanoparticles are often less susceptible to nonspecific binding than molecular fluorophores. This nonspecific binding can modify the optical properties of the fluorophore and the function of cellular proteins [114]. In this review, we will discuss the pre-clinically tested fluorescent agents according to this distinction. We will discuss nanoparticles and fluorophores bound to epidermal growth factor receptor (EGFR) targeting peptides in a separate section.

Pre-clinically, 18 studies evaluated molecular fluorophores [2, 4, 7, 18, 20, 21, 32, 43, 45, 55, 61, 77, 91, 104, 111, 112, 116] and 12 studies evaluated nanoparticles [10, 19, 46–48, 51, 68, 73, 83, 90, 108, 121]. Four of these 30 studies evaluated fluorophores or nanoparticles bound to EGF or anti-EGFR antibodies [21, 48, 90, 91]. Other fluorophores included IRDye 800CW-RGD [43], Cy5-SBK2 [7], Cy3-AS1411-TGN [61], cyclic-RGD-PLGC (Me) AG-ACPP [18], CH1055 [4], CLR1502 [104], anti-TRP-2 labeled with Alexa fluor 488 or 750 [32], motexafin gadolinium [77], BLZ-100 [8], Angiopep-2-Cy5.5 [116], DA364-Cy5.5 [55], PARPi-Fl [45], chlorotoxin:Cy5.5 [111], PEG-Cy5.5 [2], GB119-Cy5 [20] and cetuximab-IRDye 800CW [112]. Other nanoparticles included quantum dots [10, 46], iron oxide nanoparticles [51, 108, 121], polymer based nanoparticles [19, 47, 73], upconversion nanoparticles (UCNPs) [68], and liposomal nanocarriers [83].

### Molecular fluorophores

Eighteen papers described molecular fluorophores with molecular (15), metabolic (one), and passive (two) targeting mechanisms [2, 4, 7, 8, 18, 20, 21, 32, 43, 45, 55, 61, 77, 91, 104, 111, 112, 116]. Fluorophores conjugated to the integrin-targeting peptide RGD (IRDye 800CW-RGD) [43] or the protein tyrosine phosphatase mu-targeting peptide SBK2 (Cy5-SBK2) [7] showed a TNR of 16.3–79.7 and 11.7–19.8, respectively, dependent on the glioma cell line being observed. Cy5-SBK2 was tested in vivo and labeled invading tumor cells up to 3.5 mm away from the tumor margin. Molecular targeting peptides can be combined to form dual targeting probes. Targeting peptide AS1411 labeled with Cy3 showed a significantly higher uptake in glioma cells when combined with the BBB targeting peptide TGN [61]. Dual targeting of integrin and matrix metallo-proteinase (MMP-2) showed in vivo a TNR of 7.8 and in vitro an improved uptake compared to integrin and MMP targeting alone [18].

A metabolic targeting agent is the alkylphosphocholine analog (CLR1502). This was compared with 5-ALA in a mouse model, showing a significant higher TNR (9.28 vs. 4.81) [104].

Two passive targeting fluorophores were identified [4, 77]. A mouse study showed that the CH1055 molecule has a maximal TNR of 5.50 ± 0.36. The authors speculate that, in the future, this molecule could also be conjugated to anti-EGFR affibodies to increase the TNR [4]. Furthermore, motexafin gadolinium was shown to be a feasible marker for gliomas in a rat glioma model both with optical imaging and on T1 MRI [77].

### Nanoparticles

Twelve papers evaluated nanoparticles in a preclinical setting with molecular (eight studies), metabolic (two), and passive (two) targeting mechanisms [10, 19, 46–48, 51, 68, 73, 83, 90, 108, 121]. Quantum dots (QDs) are nanoparticles constructed from semiconducting nanocrystals and can function as fluorescent ‘dye’ due to their optical properties. Quantum dots have a tunable emission wavelength based on the diameter and stable fluorescence activity. They can be used as imaging or tumor-targeting agents, and specific peptides coated on the surface can modify their function [76].

QDs coated with RGD peptides (QD-RGDs) specifically target integrin molecules expressed by GBM cells. In vivo, fluorescence imaging of QD-RDGs showed a TNR of 4.42. This was significantly higher than for QDs without RDGs coated on their shell [10]. The peptide F3, which targets the tumor cell surface receptor nucleolin, enhances uptake of the fluorescent polyacrylamide nanoparticles in glioma cells by a factor of 3.1 compared to nanoparticles without F3 [73]. One study investigated FGS in mice with selective porphyrin-based nanostructure mimicking nature lipoproteins (PLP). In vivo confocal microscopy showed tumor delineation at the cellular level. FGS resulted in minimal residual tumor cells in the resection cavity [19]. Dual targeting upconversion nanoparticles (nanoparticles that are capable of absorbing two or more low-energy photons and emitting one high-energy photon) were labeled with angiopeptide-2 and PEG (ANG/PEG-UCNPs) to cross the BBB and target GBM cells in mice. Due to their bimodal imaging properties, ANG/PEG-UCNPs can be used for MRI diagnosis and fluorescence imaging for surgery [68]. Magnetic ironoxide nanoparticles use these bimodal imaging properties as well. An iron oxide nanoparticle labeled with polyethylene glycol-block-polycaprolactone (PEG-b-PCL) and the glioma-targeting ligand lactoferrin (Lf), showed a TNR of 3.8 in a mouse model [121]. Molecular targeting with lactoferrin is also performed with a polymer-based nanoparticle [47].

Cross-linked iron oxide (CLIO) labeled with Cy5.5 is a metabolic targeting nanoparticle that is internalized and accumulated in tumor cells within a maximum of 24 h after injection [51, 108]. Uptake of CLIO-Cy5.5 was also seen in microglia and macrophages at the tumor boarder, resulting in an overestimation of fluorescent enhancement beyond the tumor border between 2 and 24 μm in mice and rat models. No uptake was seen in neurons [108].

Evans Blue (EB) is a passive fluorescent agent that falsely stains healthy tissue due to diffusion. EB capsuled in a liposomal nanoparticle (nano-EB), however, showed a sensitivity and specificity in discriminating tumor from brain tissue of 89 and 100%, respectively [83]. Nano-EB did not stain healthy brain tissue, but underestimated the true margin on the order of tens to hundreds of micrometers, as reported in a rat study. High-dosed QDs coated with polyethylene glycol (PEG) are phagocytized by tumor-induced inflammatory cells (macrophages and microglia) in the tumor border, but not by tumor or brain cells. A study showed that by using QD-PEGs, the tumor margin and satellite lesions could be visualized in vivo in rats [46].

### Anti-EGFR or anti-EGF

Four preclinical studies evaluated anti-EGFR antibodies or EGF labeled with a fluorescent compound to discriminate tumor cells from adjacent brain tissue [21, 48, 90, 91]. Epidermal growth factor receptor (EGFR) is a cell-surface receptor overexpressed in many cancer types, including glioma. Gliomas express the wild-type or mutated forms of EGFR, including the GBM specific EGFRvIII. In a mouse model, glioma cells were injected in the brain and 2 weeks later nanoparticles (gold nanorods, GNR) labeled with anti-EGFR antibodies were injected intravenously. Post-mortem imaging of their brain showed a strong absorption in malignant tissue areas [90]. In a combined human and animal ex vivo study, labeling quantum dots (QDs) with EGF and anti-EGFR antibodies visualized individual tumor cells with confocal imaging reaching a TNR as high as 1000, even for LGGs. QDs bound to a combination of EGF and several EGFR antibodies were able to target mutated forms of EGFR as the GBM specific EGFRvIII [48]. In vivo imaging with MRI- fluorescence molecular tomography (MRI-FMT) of mice injected with IRDye 8000CW labeled EGF, showed a 100% sensitivity and specificity in distinguishing mice with EGFR (+) tumor cell lines from EGRF (−) tumor cell lines or control mice. Histological accuracy in distinguishing brain and tumor tissue was not calculated, however [21]. In a recent mouse study, the smaller anti-EGFR affibody protein (±7kDA) had a significantly higher concentration in the tumor periphery than the full antibody (±150 kDa) [91]. Molecular targeting of EGFR is a promising development in FGS; however, it is dependent on the expression of EGFR in tumor cells.

## Discussion

Various fluorescent agents have been studied for use in glioma surgery, of which 5-ALA, ICG, fluorescein, hypericin, AFL-HSA, and endogenous spectroscopy have been tested clinically (Table 3).

**Table 3:** Overview of clinically tested targeting agents

Agent	Excitation (nm)	Emission (nm)	Mode of targeting	GTR (%)	Survival (months/%)	Adverse effects	Remark
5-ALA	375–440	640–710	Metabolic	65 vs. 35^a^	- 12–15 vs. 6–14^a^ - PFS: 5–9 vs. 4–5^a^ - 6-PFS: 41–46% vs. 21–28%^a^	- Phototoxicity, higher rate of deterioration at 48 h	Applicable with confocal microscopy and PDT
Fluorescein	460–500	540–690	Passive	80–100 vs. 30– 55	- 11–15 vs. 10–13 - PFS: 7.4 vs. 5.4	- Coloring of skin, mucosa, and urine - Anaphylactic reactions	Applicable with confocal microscopy
ICG	778	700–850	Passive	–	–	Anaphylactic reactions	Applicable with confocal microscopy
Hypericin	415–495	590–650	Passive	–	–	No side effects observed	Application with PDT
AFL-HSA	495	535	Passive	69	–	No side effects observed	–
Endogenous (spectroscopy)	337	360–750	Endogenous	–	–	No side effects observed	–
Endogenous (multiphoton tomography)	700–1000	Dependent on excitation intensity	Endogenous	–	–	–	Destruction of single cell in 3D matrix (rat study)

*5-ALA* δ-aminolevulinic acid, *AFL-HAS* 5-aminofluorescein bound to human serum albumin, *GTR* gross-total resection, *HGG* high-grade glioma, *ICG* indocyanine green, *LGG* low-grade glioma, *nm* nanometer, *PDT* photo-dynamic therapy, *PFS* progression-free survival, *Sens* sensitivity, *Spec* specificity, *TNR* tumor-to-normal ratio; − : not specified

^a^Data from RCTs

^b^Data from a meta-analysis including only prospective studies

The three RCTs demonstrated that the use of 5-ALA-based FGS results in improved extent of resection in FGS for glioma [99], and improved PFS [26, 101]. Observational cohort studies suggest that the use of fluorescein increases the rate of GTR as well [11, 52, 92], and that it has a positive effect on PFS [11]. To date, the evidence for effectiveness of clinically tested fluorescent agents other than 5-ALA has been based on only observational cohort studies and case series. Selection bias is a major factor influencing the results in these studies. A direct comparison between 5-ALA and other fluorescent agents is therefore not possible and would require additional, specifically designed studies, however.

Methodological heterogeneity reduces comparability of the studies. Several of the clinical 5-ALA studies specifically included gliomas in eloquent areas, which could have resulted in a lower GTR rate, PFS, and overall survival compared to gliomas in surgically favorable locations [22, 31, 89]. In future studies, parameters such as tumor localization should be included so that relevant corrections can be made. 5-ALA but also fluorescein and ICG have been evaluated in combination with additional intraoperative tools to increase the visualization of the tumor margin and the extent of resection, thereby reducing the comparability of different studies. Different timing and dose of the fluorescent agent add to the differences between the studies as well. Fluorescein, for example, was administered intravenously at the time of anesthesia induction [23] or opening of the dura mater [52] with dosage regimens ranging from 3 mg/kg [23] to 20 mg/kg [52]. Also, it is essential that a more standard definition of GTR is used. In most of the selected studies, GTR was defined as absence of contrast enhancement on post-operative MRI [27]. Other definitions included a reduction of more than 98% of the tumor volume based on volumetric measures [31], or less than 0.175 cm^3^ contrast enhancement on the post-operative MRI [26]. Instead of GTR, some authors report volumetric differences between pre- and post-operative MRI [15, 25]. Furthermore, the timing of the post-operative MRI varied between the studies from less than 24 h [52], less than 72 h [99], less than 1 week [11] to up to even 1 month [92] after surgery. Often, no details were provided by whom the post-operative MRIs were evaluated and if they were blinded to the procedure performed [11]. This variety in timing, reading of the images, and blinding affect the quality of assessment and comparability of the reported GTR rates among all studies.

Reported sensitivities and specificities of the various agents to distinguish brain from tumor tissue vary greatly between the included studies. Observational studies suggest that all clinically tested exogenous agents had a lower histological accuracy in LGGs compared to HGG [28, 33, 35, 86, 109, 110, 113]. In contrast, endogenous fluorophores showed a higher histological accuracy in LGGs compared to HGGs [9, 118]. However, this outcome measure is very susceptible to bias given the lack of uniform agreement on what samples should be studied. The results are very dependent on the number, timing, and location of biopsy samples taken during surgery. These details are often lacking or described in a non-reproducible and non-comparable fashion.

Pre-clinically, many fluorescent agents with different (more targeted) mechanisms of action are being developed and tested for FGS for glioma (Table 4). Agents targeting EGFR (vIII) show promising histological accuracy results [48]. It should be noted, however, that the included studies were extremely heterogeneous in study design. Furthermore, pre-clinically tested agents were not used as guidance during surgery in patients but mostly assessed on their histological accuracy in ex vivo and in vitro models. A comparison between pre-clinically and clinically tested agents based on these reports is therefore not possible.

**Table 4:** Overview of pre-clinically tested targeting agents

Agent	Fluorescent compound	Emission peak (nm)	Mode of targeting	Histological accuracy
IRDye 800CW-RGD	Fluorophore	794	Molecular	TNR 16.3–79.7
Cy3-AS1411-TGN	Fluorophore	570	Molecular	–
Cy5-SBK2	Fluorophore	670	Molecular	TNR 11.7–19.8
Cyclic-RGD-PLGC (Me)AG-ACPP	Fluorophore	670	Molecular	TNR 7.8
Anti-TRP-2-Alexa fluor 488 or 750	Fluorophore	519 or 775	Molecular	–
CLR1502	Fluorophore	778	Metabolic	TNR 9.28 (vs. 4.81 in 5-ALA)
CH1055	Fluorophore	1055	Passive	TNR: 5.50 ± 0.36
Motexafin gadolinium	Fluorophore	750	Passive	–
Cetuximab-IRDye 800CW	Fluorophore	794	Molecular	–
EGF – IRDye 800CW	Fluorophore	794	Molecular	–
Anti-EGFR affibody protein – IRD 800CW	Fluorophore	794	Molecular	–
PEG-Cy5.5	Fluorophore	665	Passive	–
BLZ-100	Fluorophore	700–850	Molecular	–
PARPi-FL	Fluorophore	525	Molecular	–
DA364-C5.5	Fluorophore	694	Molecular	–
GB119-Cy5	Fluorophore	665	Molecular	–
Angiopep-2-Cy5.5	Fluorophore	694	Molecular	TNR 1.6
Chlorotoxin:Cy5.5	Fluorophore	694	Molecular	–
CLIO-Cy5.5	Nanoparticle	694	Metabolic	–
QD-RGD	Nanoparticle	705	Molecular	TNR 4.42^a^
QD-PEG	Nanoparticle	705	Passive	–
Polyacrylamide NP – F3	Nanoparticle	Dye dependent	Molecular	n.q.^b^
Lf-MPNA nanogel – Cy5.5	Nanoparticle	694	Molecular	–
Liposomal EB nanocarrier	Nanoparticle	680	Passive	sens 89% spec 100%
ANG/PEG-UCNPs	Nanoparticle	800	Molecular	–
Lf-SPIO - Cy5.5	Nanoparticle	694	Molecular	TNR 3.8^c^
PLP – Porphyrine	Nanoparticle	645–730	Molecular	–
QD – Anti-EGFR antibody & QD-EGF	Nanoparticle	635–675	Molecular	TNR 200–1000
GNR – Anti-EGFR antibody	Nanoparticle	600–1200	Molecular	–

*ACPP* activatable cell-penetrating peptide, *ANG* angiopeptide, *AS1411* glioma-targeting aptamer, *BLZ-100* indocyanine green conjugated to chlorotoxin, *CLIO* cross-linked iron oxide, *Cy3* cyanine3, *Cy5.5* cyanine5.5, *EB* Evans Blue, *EGF (R)* epidermal growth factor (receptor), *GNR* gold nano rods, *Lf* lactoferrin, *MPNA* poly (*N*-isopropylacrylamide-co-acrylic acid), *n.m*. nanometer, *n.q*. not quantified, *NP* nanoparticle, *PEG* polyethylene glycol, *PLP* porphylipoprotein, *QD* quantum dots, *RGD* integrin-targeting peptide, *SBK2* protein tyrosine phosphatase mu-targeting peptide, *Sens* sensitivity, *Spec* specificity, *SPIO* superparamagnetic iron oxide nanoparticle, *TGN* blood–brain barrier targeting peptide, *TNR* tumor-to-normal ratio, *TRP* tyrosinase-related protein, *UCNPs* upconversion nanoparticles, −: not specified

^a^Significantly higher TNR compared to mice injected with QDs without RGD peptide coating

^b^Significantly higher uptake in glioma cells than MPNA nanogels without lactoferrin labeling

^c^Significantly higher TNR compared to mice injected with Cy5.5-SPIO without lactoferrin labeling

Previously, three excellent meta-analyses evaluated the effect FGS on GTR rate and survival [27, 103, 120]. All three included HGG patients only, however, two of which were limited to 5-ALA alone [27, 120] and one to 5-ALA, fluorescein, and hypericin [103]. One paper comprehensively reviewed only the clinically tested exogenous agents though [57]. A more recent systematic review focused on pre-clinically tested molecular targeting agents for visualizing GBM tissue [17]. This systematic review does not include all pre-clinically tested agents, however. To our knowledge, this is the first paper that systematically reviews all existing literature on all pre-clinically and clinically tested contrast agents for FGS in low- and high-grade gliomas.

### Challenges in evaluating fluorescent agents and future research

The evaluation of fluorescent agents has many challenges. For the purpose of this review, we chose the rate of GTR, PFS, overall survival, and histological accuracy (sensitivity, specificity, TNR) as outcome measures, because these are the most frequently reported outcome measures among these studies. This does not necessarily mean that these are the most appropriate measures to evaluate fluorescent agents. As indicated by Stummer et al. in 2011, the 5-ALA study was designed for testing the efficacy and safety of 5-ALA as a surgical tool and a diagnostic drug for glioma surgery. In the process of developing the 5-ALA study, the European Medical Evaluations Agency advised to test the agent in a prospective, randomized setting according to the same standards as those for cytotoxic drugs [101]. The study of Schebesch et al. in 2013 demonstrated that FGS can also be evaluated by classifying them as ‘helpful’ or ‘not helpful’ by the operating neurosurgeon [86]. Even though this might be less objective than the outcome measures included in this review, subjective outcomes like this are nevertheless very helpful for the practicing neurosurgeon.

Furthermore, GTR rate and PFS are radiological outcome measures used as indicators for clinical outcome. Overall survival, neurological symptoms, need for re-resection or adjuvant therapy, and quality-of-life assessments would be examples of other, perhaps more direct clinical outcomes that could be used, although these may be more difficult to assess and quantify. If GTR and PFS are to be used as indicators for clinical outcome, what would be the cut-off value to pursue? Residual tumor tissue on the post-operative MRI is shown to result in a decrease in overall survival, but the absolute differences in median post-operative tumor volume were very small (0 cm^3^ in the 5-ALA group vs. 0.5 cm^3^ in the control group) in the two RCTs of Stummer [99, 101]. Defining to what extent tumor resection is clinically relevant helps not only in standardizing the definition of GTR for comparison between studies but also aids in balancing maximal cytoreduction and preservation of functional outcome.

Well-designed trials to evaluate the safety and effectiveness of different fluorescent agents before introduction in the clinic are essential. We recognize, however, that RCTs for this purpose offer specific challenges, and applaud the efforts by Stummer et al. in evaluating a diagnostic and surgical tool according to therapeutic standards. Other challenges to be overcome include the impossibility of a double-blind study design in this context, as the surgeon cannot be blinded for the use of fluorescent agents, the potential learning curve in the clinical application of these products, and inter- and intra-surgeon variability. Despite these challenges, the results of both pre-clinical and clinical studies on fluorescent agents for use in glioma surgery provide a growing body of evidence of both effectiveness and safety that will likely continue to develop as these products are transitioned more frequently into clinical practice.

## Conclusions

In FGS for glioma, fluorescent agents should be easy to apply, safe to use, and tumor-specific. The fluorescent signal should be strong and easy to detect. Currently, 5-ALA is the only agent that has been tested in a multi-center RCT and has been approved for clinical use in certain parts of the world. Other clinically tested exogenous agents for FGS for glioma include fluorescein, ICG, AFL-HSA, and hypericin. Despite their contributions to GTR, due to their non-specific mechanism of action, preclinical research has shifted away from these products and towards molecular targeting (e.g., anti-EGFR). As histological accuracy increases with the improvement of fluorescent agents, there will be emerging interest in visualization at the cellular level with imaging systems like confocal microscopy. Currently, direct comparisons between the various agents are not possible and would require additional studies. Future studies could make such comparisons possible by using a more standardized, uniform design, with improved definitions of GTR and a broader set of outcome measures.

## Abbreviations

5-ALA: 5-aminolevulinic acid
BBB: Blood–brain barrier
EGFR: Epidermal growth factor receptor
FGS: Fluorescence-guided surgery
GTR: Gross total resection
HGG: High-grade glioma
ICG: Indocyanine green
LGG: Low-grade glioma
MRI: Magnetic resonance imaging
NIR: Near-infrared
PFS: Progression-free survival
PpIX: Protoporphyrin
QD: Quantum dot
TNR: Tumor-to-normal ratio
